# Anterior cervical discectomy and fusion versus posterior cervical foraminotomy for the treatment of single-level unilateral cervical radiculopathy: a meta-analysis

**DOI:** 10.1186/s13018-020-01723-5

**Published:** 2020-06-01

**Authors:** Wenguang Fang, Lijun Huang, Feng Feng, Bu Yang, Lei He, Guizhong Du, Peigen Xie, Zihao Chen

**Affiliations:** 1 Orthopedic Center, The Sixth People’s Hospital of Huizhou, No. 2 Aimindong Road, Huizhou, 516211 Guangdong China; 2 Department of Spine Surgery, The Third Affiliated Hospital of Sun Yat-sen University, No. 600 Tianhe Road, Guangzhou, 510630 Guangdong China

**Keywords:** Cervical radiculopathy, Anterior cervical discectomy and fusion, Posterior cervical foraminotomy, Meta-analysis

## Abstract

**Background:**

To compare the effectiveness and safety of anterior cervical discectomy and fusion (ACDF) with posterior cervical foraminotomy (PCF) for patients diagnosed with single-level unilateral cervical radiculopathy.

**Methods:**

Relevant studies comparing ACDF with PCF for cervical radiculopathy were searched in an electronic database. After data extraction and quality assessment of included studies, a meta-analysis was done by using the RevMan 5.3 software. The random effects model was used if there was heterogeneity between studies; otherwise, the fixed effects model was used.

**Results:**

A total of 3 randomized controlled trials (RCT) and 12 retrospective studies including 52705 patients were included in the meta-analysis. There were no significant differences in Neck Disability Index (NDI), Visual Analog Scale (VAS), and patients’ satisfaction (*P* > 0.05) between treatment groups. The complication rate of the PCF group was equivalent compared with the ACDF group (*P* = 0.60), but the reoperation rate following PCF was on the higher side (*P* = 0.02). Data analysis also showed that the PCF group was associated with shorter operation time (*P* = 0.001) and shorter length of hospital stay (*P* = 0.002).

**Conclusions:**

Among patients with single-level unilateral cervical radiculopathy, PCF has comparable effectiveness and complication rate compared with ACDF. It seems that PCF is a sufficient alternative procedure with shorter operation time, shorter length of hospital stay, and less total hospital cost for the treatment of cervical radiculopathy. However, the higher reoperation rate following PCF should be also taken into consideration.

## Background

Cervical radiculopathy is defined as pain in a radicular pattern in one or both upper extremities related to compression and/or irritation of one or more cervical nerve roots secondary to disc herniation or lateral foraminal stenosis [1]. Various degrees of sensory, motor, and/or reflex change in the upper extremity are the common signs and symptoms. Surgical intervention is suggested for rapid pain relief for cervical radiculopathy when compared with non-surgical treatments [2, 3].

Anterior cervical discectomy and fusion (ACDF) is considered to be the “gold standard” surgical intervention by many surgeons and is proved to be an effective treatment for cervical radiculopathy [4, 5]. However, the disadvantages of ACDF, including adjacent segmental diseases, pseudoarthrosis, instrument-related compilation, and ventral approach-related complications should be also taken into account [6–8].

Posterior cervical foraminotomy (PCF) is an alternative surgical approach for the treatment of cervical radiculopathy. The posterior approach can avoid the ventral approach-related complications, such as postoperative dysphagia, hematoma, and recurrent laryngeal nerve palsy. Moreover, PCF can preserve the range of motion in the operated segment and impose less stress on the adjacent segment [9, 10]. However, it was reported that PCF is associated with a higher incidence of reoperation [10]. Recently, PCF can be performed through a minimally invasive approach (MI-PCF) using tubular retractor or endoscopy, and the effectiveness of MI-PCF is considered to be equivalent to open PCF [11].

Many studies have compared the outcomes of ACDF with PCF for the treatment of cervical radiculopathy in recent years. However, controversial outcomes were reported on those studies and no consensus can be reached till now. Although 2 previous meta-analyses comparing MI-PCF and ACDF have already published in the last 2 years, both of them have limitations in methodology. Sahai et al. conducted a meta-analysis of MI-PCF outcome data from 14 non-comparative studies, and the pooled outcomes were compared to those of ACDF cohorts obtained from two previously published studies [12]. However, great selecting bias may exist because all of the MI-PCF data came from non-comparative studies without ACDF controlled group. Another meta-analysis of randomized controlled studies was published by Gutman et al. in 2018, but only 1 RCT was included in that study and hence meta-analyses for all clinical outcomes could not be performed indeed [13]. Therefore, we performed a meta-analysis of recent comparative studies to compare the clinical outcomes, complication, and reoperation rate between these two approaches.

## Methods

### Inclusion and exclusion criteria

Studies were included if they met the following inclusion criteria: (1) randomized or nonrandomized comparative studies, (2) patients who diagnosed with single-level unilateral cervical radiculopathy, (3) comparative data between ACDF and PCF were available, and (4) sample size was bigger than 10.

Studies were excluded according to the following exclusion criteria: (1) patients with multilevel or bilateral cervical radiculopathy; (2) patients with cervical myelopathy, instability, trauma, tumor or infection; and (3) patients who had previous cervical surgery.

### Search strategies

A computerized search was conducted on electronic databases of PubMed, Scopus, and the Cochrane Library. The following search terms were used: “anterior,” “discectomy,” “fusion,” “posterior,” “foraminotomy,” “laminoforaminotomy,” “keyhole,” and “cervical,” with combinations of the Boolean operators “AND” and “OR.” The date of publication of studies was limited from January 1990 to July 2019. References of relevant review articles were checked additionally to identify more underlying studies.

### Data extraction

Data were extracted from included studies by two investigators independently. Disagreements were solved by further discussion. The following data were extracted: demography information, clinical outcomes, amounts of complication and reoperation, operation time, intraoperative blood loss, length of hospital stay, and total hospital costs. To assess the clinical outcomes of cervical radiculopathy, the Neck Disability Index (NDI) and visual analog scale (VAS) are recommended [1]. The extracted data had been rechecked to ensure accuracy.

### Quality assessment

The Newcastle-Ottawa Scale (NOS) [14] was used for the quality assessment of non-randomized comparative studies, and the NOS is recommended by Cochrane Handbooks version 5.10 [15]. The NOS uses a “star system” (maximum of nine stars) to assess the following three aspects: the selection of study groups, the comparability of study groups, and the ascertainment of the outcome of interest for cohort studies. The qualities of the studies were assessed by two investigators. RCTs and those non-randomized studies with six or more stars were considered to be of relatively high quality. After quality assessment, those low-quality studies would be excluded from the meta-analysis.

### Data analysis

The Review Manager software (RevMan 5.3, The Cochrane Collaboration, Oxford, UK) was used for statistical analysis. Continuous variables were reported as weighted mean difference (WMD) and 95% confidence interval (95%CI), while dichotomous variables were reported as odds ratios (OR) and 95%CI. The random effects model was used if there was heterogeneity (*I*^2^ ≥ 50% in heterogeneity test) among studies; otherwise, the fixed effects model was used (*I*^2^ < 50% in heterogeneity test). Extracted data were entered into computer, rechecked, and analyzed by two investigators independently.

Included studies were divided into 2 subgroups according to the surgical technique applied in the PCF group: the open subgroup (ACDF VS open-PCF) [9, 16–25] and the MI subgroup (ACDF VS MI-PCF) [10, 26–28].

## Results

### Search result

**Fig. 1:**
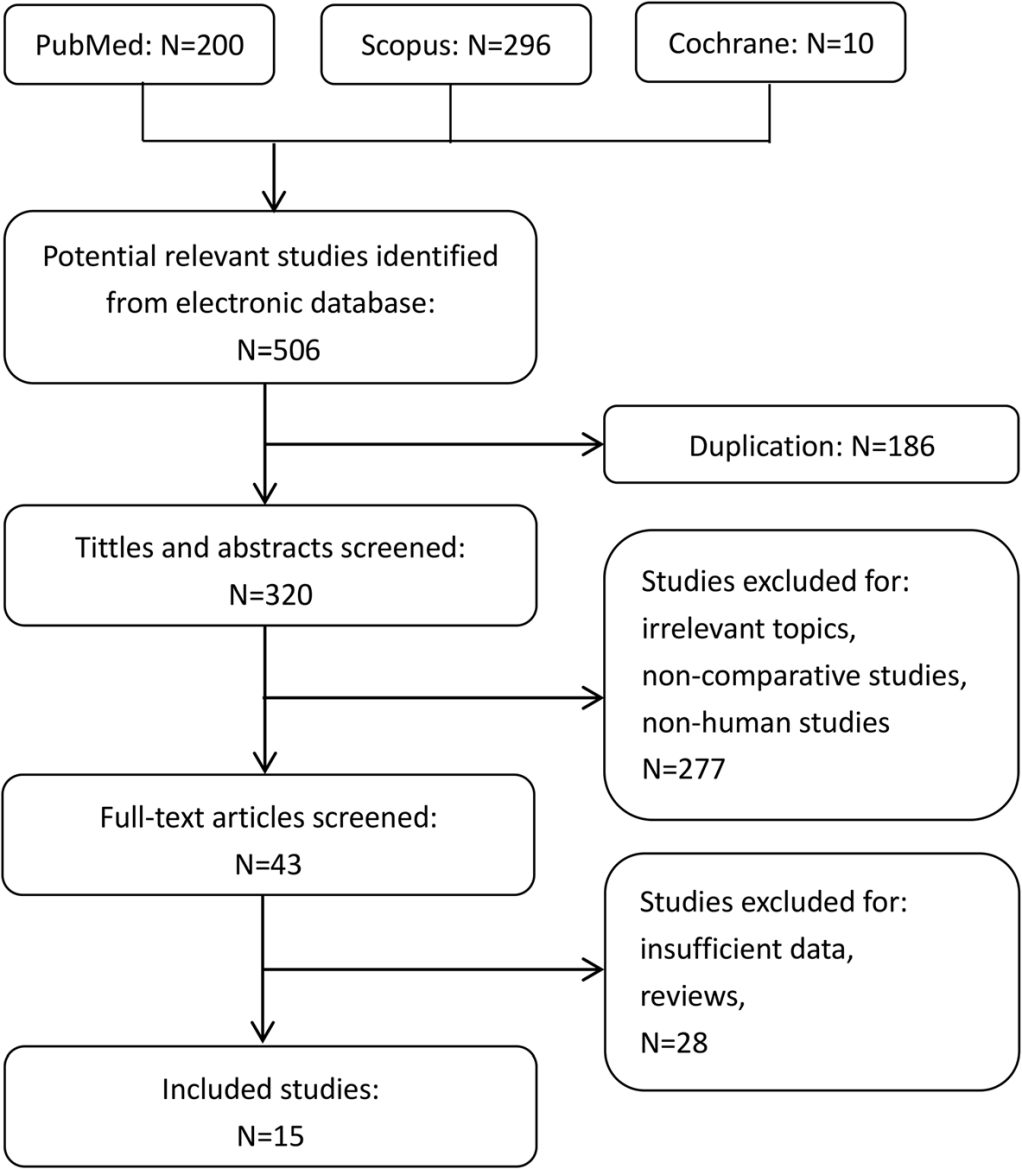
Flow diagram of study selection

This meta-analysis has been reported according the Preferred Reporting Items for Systematic Reviews and Meta-Analyses (PRISMA) statement [29]. A total of 506 relevant studies were identified from the electronic database, of which 320 studies were obtained after the removal of duplicated studies. Based on the title and abstract, 277 studies were excluded. After careful full-text evaluation of the remaining 43 studies, 15 studies including 54107 cases met the predefined inclusion criteria and were included in the final synthetic analysis (Fig. 1). Three of them were randomized controlled trials [24, 25, 28], and the other 12 studies were non-randomized comparative studies [9, 10, 16–23, 26, 27]. The basic characteristics of the included studies are shown in Table 1. Concerning baseline differences, we found that patients in the PCF group were younger (*P* < 0.00001), with significantly more male patients (*P* = 0.03). No statistical differences were found on preoperative VAS-neck, preoperative VAS-arm, and preoperative NDI score between the two groups.

**Table 1:** Basic characteristics of included studies

Studies	Study type	Country	Group	Sample size	Mean age (years)	Gender (Male)	Follow up (month)
Alvin et al. [20]	Retrospective	USA	ACDF	45	49.3 ± 9.6	28 (62.2%)	12
			open PCF	25	46.5 ± 11.2	15 (60.0%)	12
Cho et al. [9]	Retrospective	Korea	ACDF	30	49.7 ± 10.4	17 (56.7%)	61.2 ± 20.2
			open PCF	31	52.4 ± 9.6	22 (71.0%)	62.6 ± 19.9
Dunn et al. [26]	Retrospective	USA	ACDF	210	46.6 ± 6.6	95 (45.2%)	44.9 ± 10.3
			MI-PCF	49	46.9 ± 10.3	38 (77.6%)	42.9 ± 6.6
Forster et al. [18]	Retrospective	UK	ACDF	634	49 ± 12	313 (49.4%)	24
			open PCF	54	50 ± 11	17 (31.5%)	24
Herkowitz et al. [25]	RCT	USA	ACDF	17	43 (26–52)	NA	60.4 (19.2–98.4)
			open PCF	16	39 (21–50)	NA	
Korinth et al. [23]	Retrospective	Germany	ACDF	124	45.9 ± 8.2	73 (58.9%)	72.1 ± 25.9
			open PCF	168	46.9 ± 10.4	98 (58.3%)	
Lin et al. [10]	Retrospective	Korea	ACDF	55	52.5 ± 10.7	31 (56.4%)	39.5 ± 13.5
			MI-PCF	21	53.1 ± 11.9	14 (66.7%)	35.9 ± 16.6
Mansfield et al. [27]	Retrospective	USA	ACDF	79	49 (24–75)	35 (46.1%)	NA
			MI-PCF	22	49 (31–69)	12 (57.1%)	NA
Mok et al. [17]	Retrospective	USA	ACDF	816	51.2 ± 11	400 (49.0%)	NA
			open PCF	145	53.8 ± 9.3	82 (58.2%)	NA
Ruetten et al. [28]	RCT	Germany	ACDF	86	NA	NA	24
			MI-PCF	89	NA	NA	24
Scholz et al. [19]	Retrospective	Germany	ACDF	40	50 ± 10.1	20 (50.0%)	33 ± 18.4
			open PCF	67	51 ± 10.7	38 (56.7%)	47 ± 16.4
Selvanathan 2015 [21]	Retrospective	UK	ACDF	150	48	61 (40.7%)	24 ± 1.4
			open PCF	51	50	34 (66.7%)	25 ± 1.2
Tumialan et al. [22]	Retrospective	USA	ACDF	19	39.3 (24–52)	19 (100%)	18.1 (6–34)
			open PCF	19	41.4 (27–56)	17 (89.5%)	11.2 (5–24)
Wirth et al. [24]	RCT	USA	ACDF	25	41.7	14 (56.0%)	2
			open PCF	22	43.8	9 (40.9%)	2
Witiw et al. [16]	Retrospective	USA	ACDF	46147	48.5 ± 9.6	21141 (45.8%)	1
			open PCF	4851	49.7 ± 9.7	2821 (58.2%)	1

*RCT* randomized controlled trial, *ACDF* anterior cervical discectomy and fusion, *PCF* posterior cervical foraminotomy, *MI* minimally invasive, *NA* not available

### Quality assessment

Quality assessment of included studies was conducted by two investigators independently and the argument was solved by discussion. All of the 12 non-randomized comparative studies got more than 7 stars on the NOS, which meant that these studies were of relatively high quality. The results of the quality assessment are summarized in Table 2 as well.

**Table 2:** Quality assessment of non-randomized comparative studies

Study	Selection	Comparability	Outcome	Total
Alvin et al. [20]	4	2	3	9
Cho et al. [9]	3	1	3	7
Dunn et al. [26]	4	1	3	8
Forster et al. [18]	4	2	3	9
Korinth et al. [23]	4	1	3	8
Lin et al. [10]	4	1	3	8
Mansfield et al. [27]	3	1	3	7
Mok et al. [17]	4	1	2	7
Scholz et al. [19]	3	0	3	7
Selvanathan et al. [21]	3	1	3	7
Tumialan et al. [22]	3	2	3	8
Witiw et al. [16]	4	1	2	7

### Clinical outcomes

**Fig. 2:**
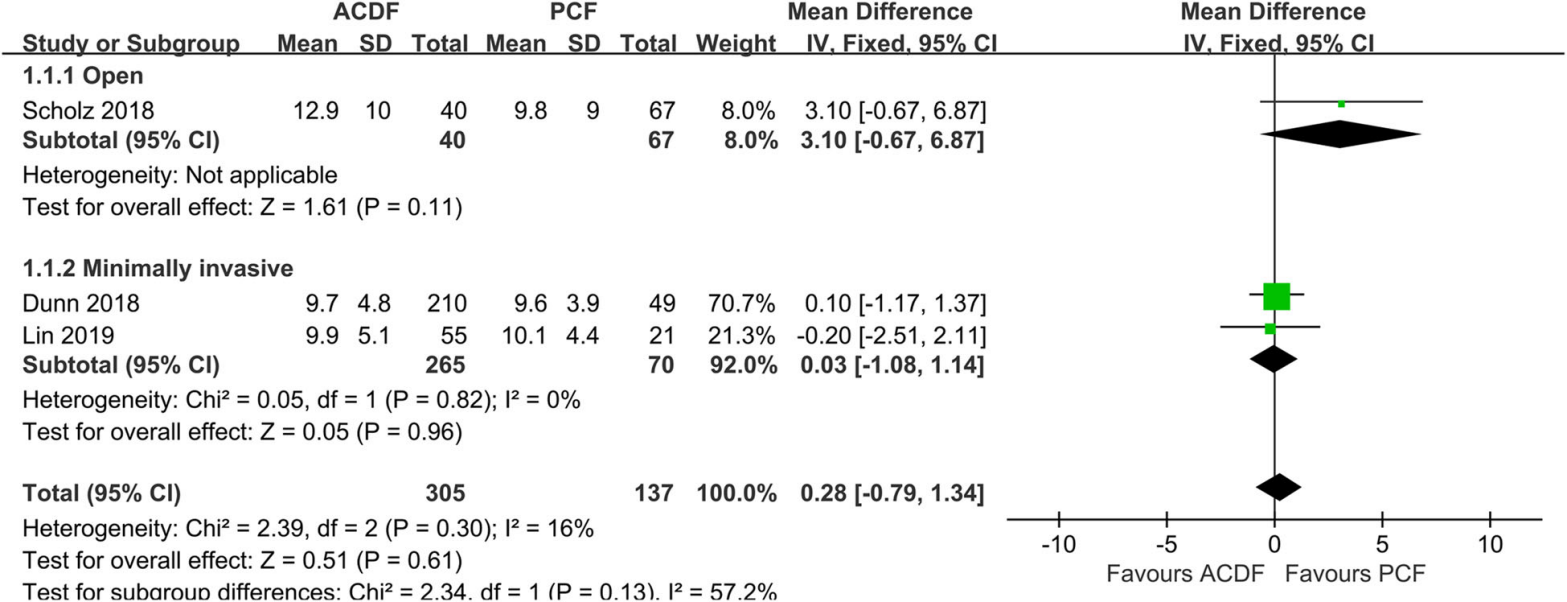
Forest plot of NDI between the ACDF group and PCF group

Data of postoperative NDI score was extracted from 3 studies [10, 19, 26]. The total postoperative NDI score in the ACDF group was comparative with those in the PCF group (*P* = 0.61, WMD 0.28, 95%CI − 0.79 to 1.34; *I*^2^ = 16%; Fig. 2). Subgroup analysis showed similar results in an open subgroup (*P* = 0.11, WMD 3.10, 95%CI − 0.67 to 6.87) and MI subgroup (*P* = 0.96, WMD 0.28, 95%CI − 0.79 to 1.34).

**Fig. 3:**
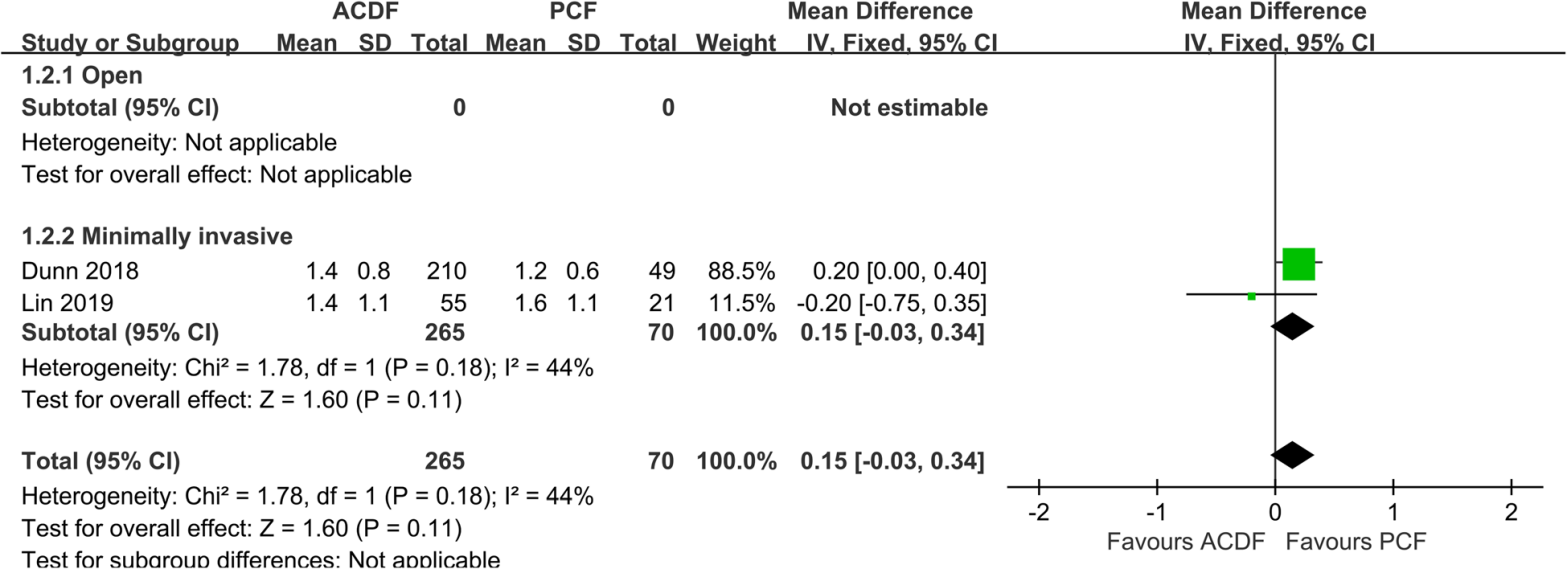
Forest plot of VAS-neck between the ACDF group and PCF group

Data of VAS-neck was only available in 2 studies [10, 26] comparing ACDF with MI-PCF. No significant difference of postoperative VAS-neck was found between the 2 groups (*P* = 0.11, WMD 0.15, 95%CI − 0.03 to 0.34; *I*^2^ = 44%; Fig. 3).

**Fig. 4:**
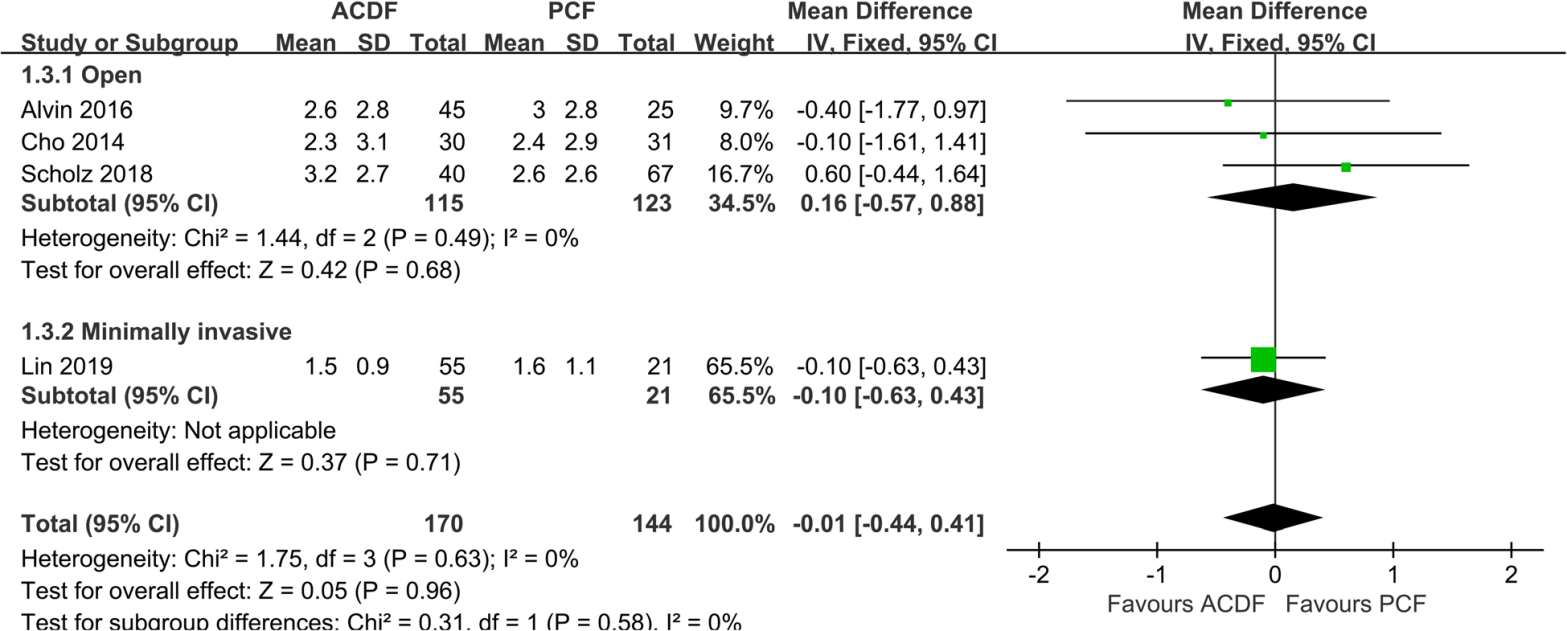
Forest plot of VAS-arm between the ACDF group and PCF group

Data of VAS-arm was available in 5 studies [9, 10, 19, 20, 26]. Since the postoperative scores of VAS-arm are not consistent with the reported *P* value in the manuscript, we excluded the data of VAS-arm from 1 study [26]. PCF achieved similar postoperative VAS-arm compared with ACDF in both open (*P* = 0.11, WMD 0.61, 95%CI − 0.14 to 1.35; *I*^2^ = 0%; Fig. 4) and MI (*P* = 0.71, WMD 0.14, 95%CI − 0.29 to 0.57) subgroup analysis.

**Fig. 5:**
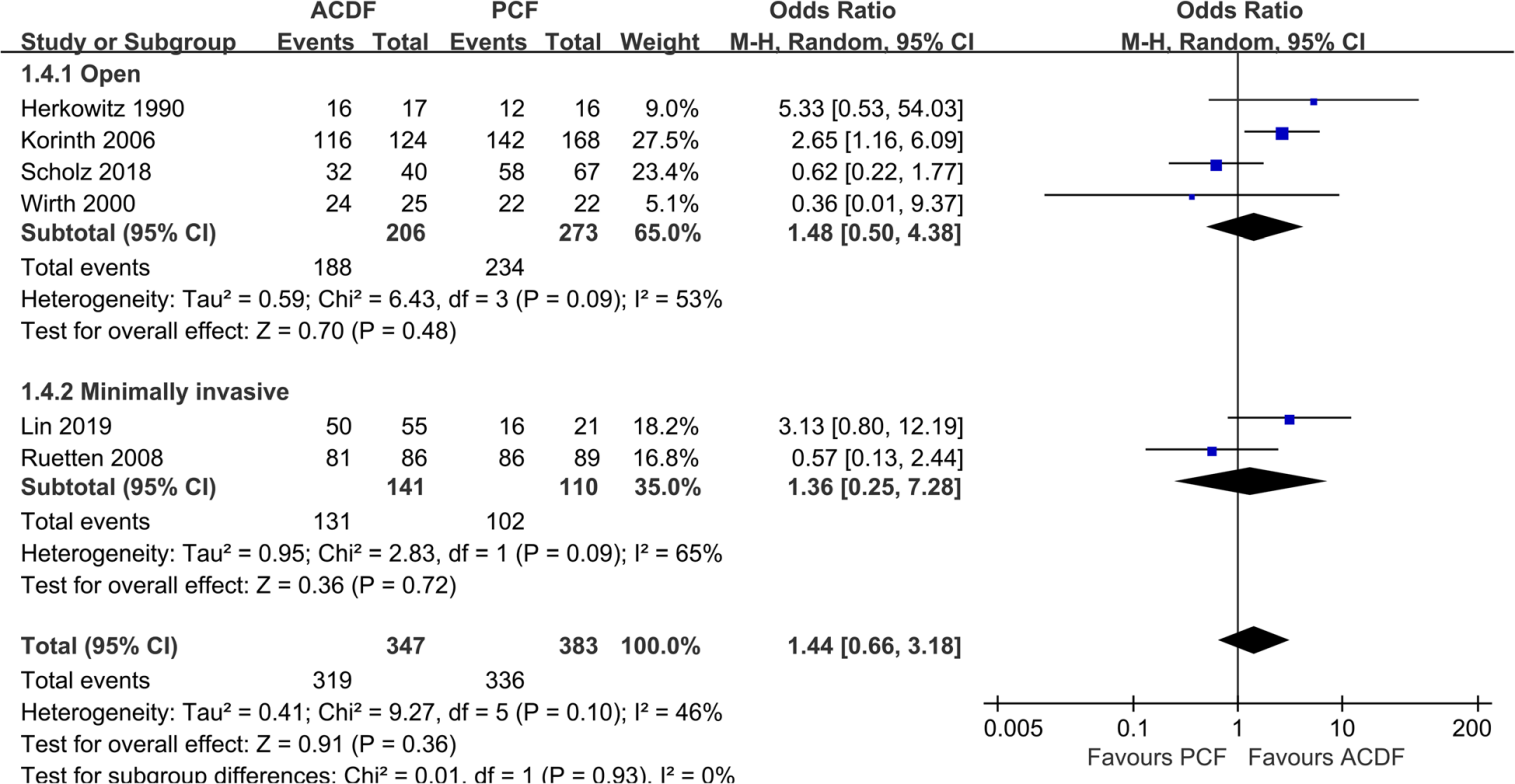
Forest plot of patient satisfaction between the ACDF group and PCF group

The degree of satisfaction (excellent or good outcomes) was reported in 6 studies [10, 19, 23–25], and there was no significant difference between the two groups (*P* = 0.36, OR 1.44, 95%CI 0.66 to 3.18; *I*^2^ = 46%; Fig. 5).

### Complications and reoperations

**Fig. 6:**
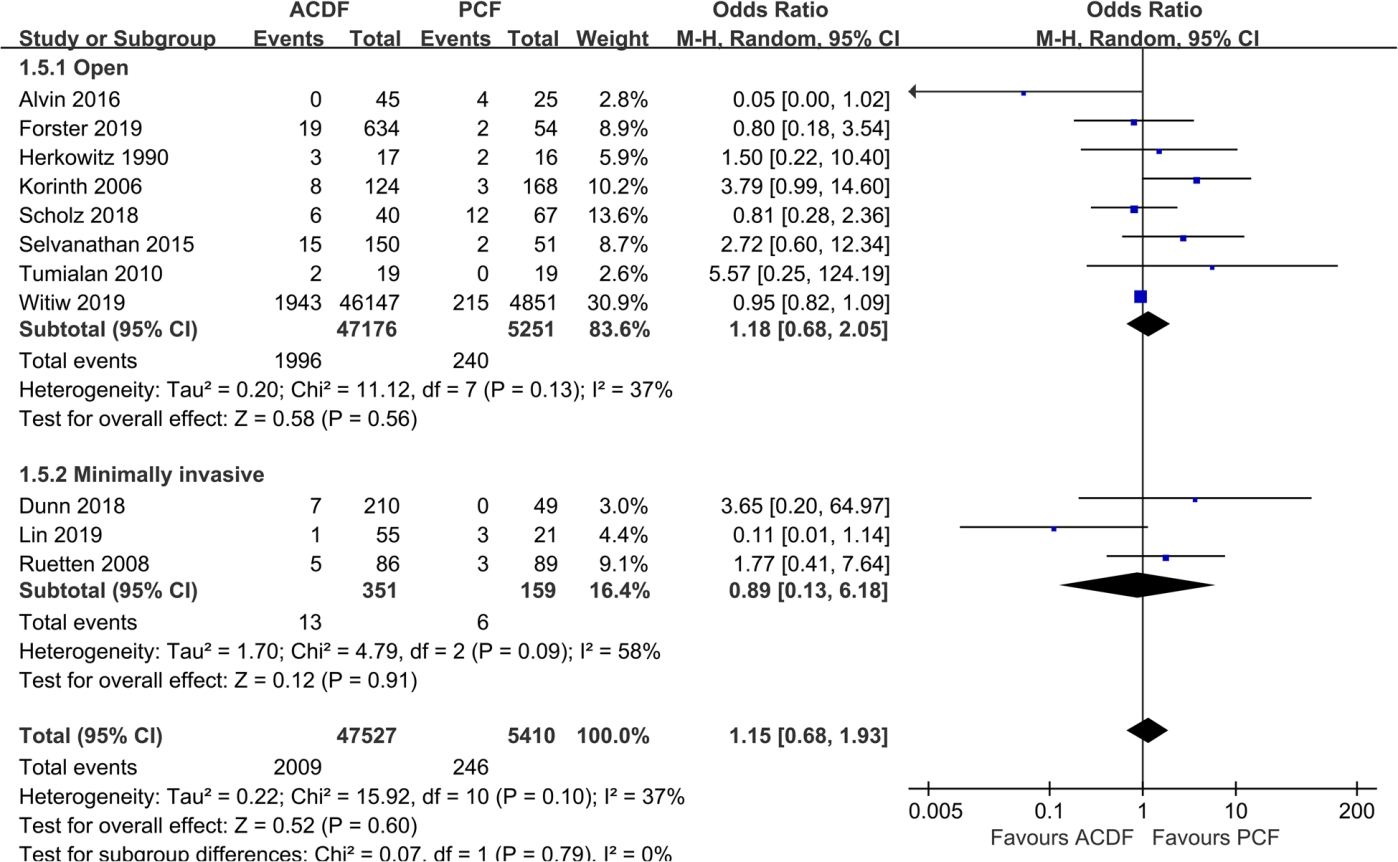
Forest plot of overall complication rate between the ACDF group and PCF group

Data of complications was available in 11 studies [10, 16, 18–23, 25, 26, 28]. According to the statistical analysis, no significant difference was found in the complication rate between the two groups (*P* = 0.60, OR 1.15, 95%CI 0.68 to 1.94; Fig. 6). Data between studies was of low heterogeneity (*I*^2^ = 37%). The total complication rate of included studies was 4.23% in ACDF group and 4.55% in the PCF group.

**Fig. 7:**
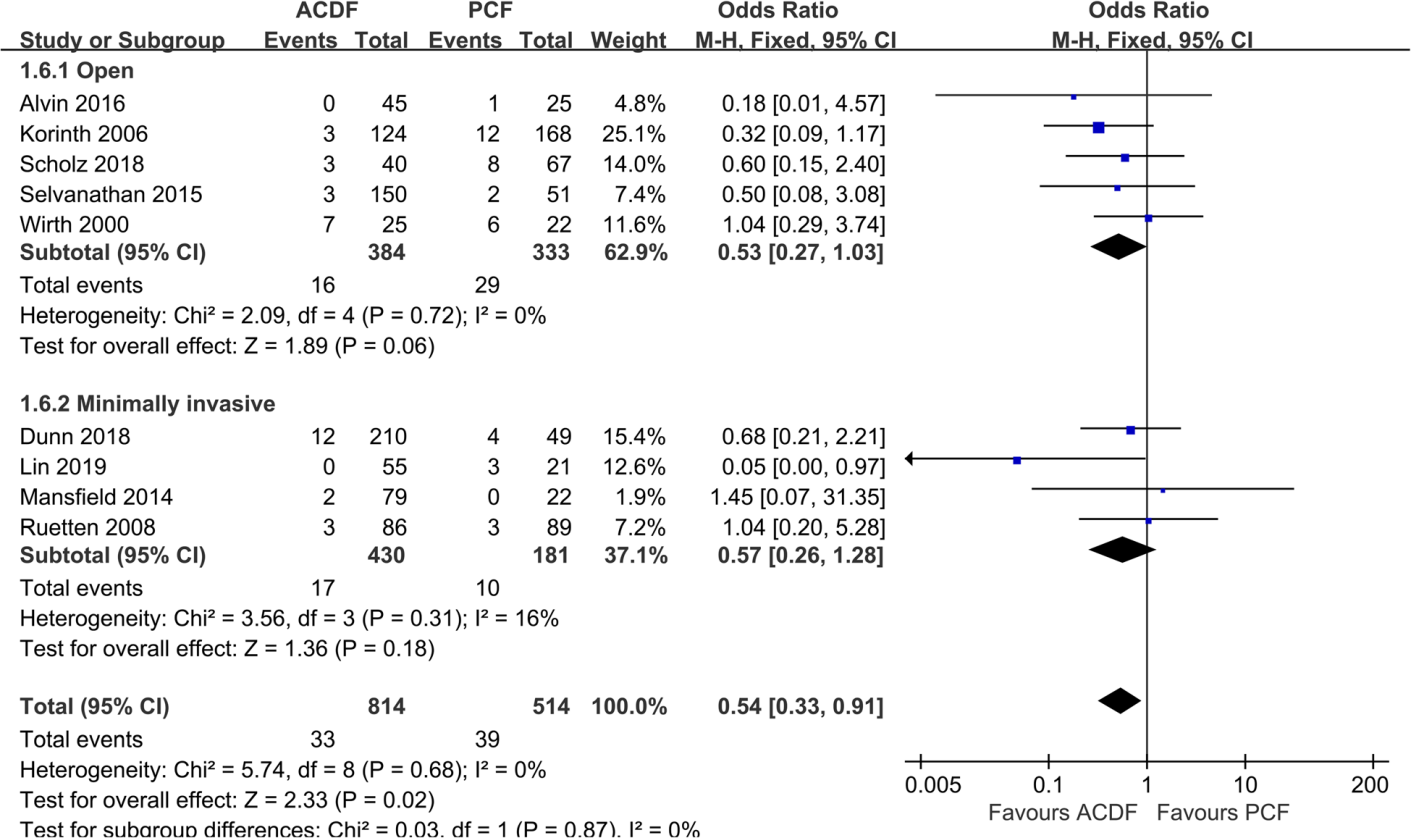
Forest plot of overall reoperation rate between the ACDF group and PCF group

There were 9 studies [10, 19, 21, 23, 24, 26–28] that offered the data of reoperation. ACDF group has a statistically lower reoperation rate than those in the PCF group (*P* = 0.02, OR 0.54, 95%CI 0.33 to 0.91; *I*^2^ = 0%; Fig. 7).

### Perioperative outcomes

**Fig. 8:**
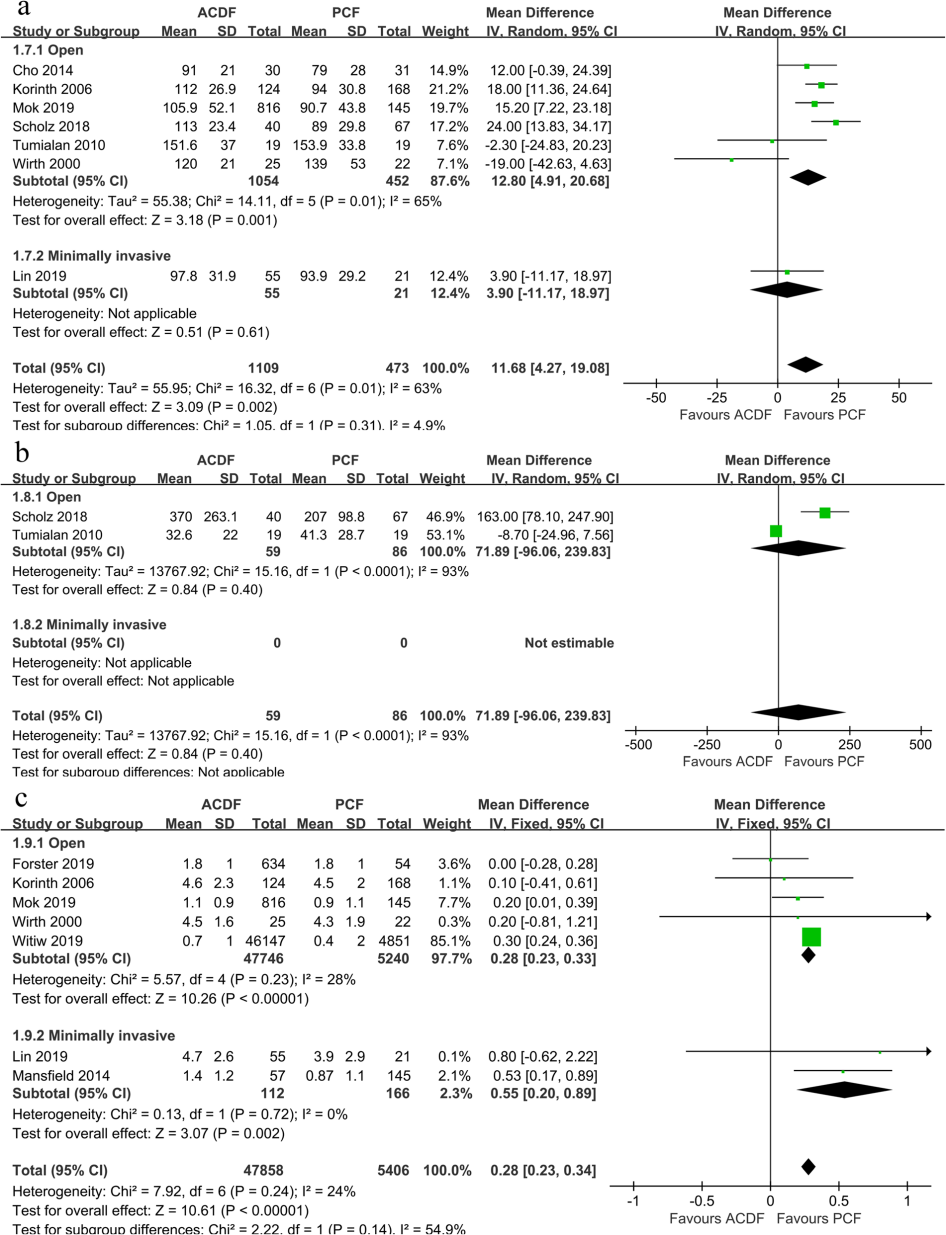
Forest plot between the ACDF group and PCF group for **a** operation time, **b** intraoperative blood loss, and **c** length of hospital stay

Seven studies reported the data of operation time, 6 in the open subgroup [9, 19, 21, 26, 27, 29] and 1 in the MI subgroup [11]. Compared to ACDF, open-PCF had shorter operation time (*P* = 0.001, WMD 12.8, 95%CI 4.91 to 20.68; *I*^2^ = 65%; Fig. 8a). Different from the result from the open subgroup, MI-PCF had similar operation time compared with ACDF (*P* = 0.61, WMD 3.90, 95%CI 4.27 to 19.08).

Only 2 studies [21, 26] in the open subgroup reported the data of intraoperative blood loss, and no significant difference was found in such data (*P* = 0.40, WMD 71.89, 95%CI − 96.06 to239.83; *I*^2^ = 93%; Fig. 8b).

Data of length of hospital was extracted from 7 studies [11, 17, 19, 20, 27, 29, 30]. The PCF group was associated with significantly shorter length of hospital than the ACDF group (*P* = 0.002, WMD 0.28, 95%CI 0.23 to 0.34; *I*^2^ = 24%; Fig. 8c).

Data of total hospital cost was reported in 3 studies [17, 22, 30]. The total hospital cost of the PCF group was significantly less than that of the ACDF group (*P* = 0.01, WMD 7063.89, 95%CI 1468.19 to 12659.60; *I*^2^ = 99%).

### Sensitivity analysis

We have conducted the sensitivity analysis to evaluate whether the relevant results would be changed when excluded the data from Witiw et al.’s study [17], because majority of the included cases were from Witiw et al.’s study (*n* = 50998), which was far more than the sum of the other studies (*n* = 3109). When the Witiw et al.’s study was excluded in the sensitivity analysis, no changes were seen in terms of the significance of each outcome. The results were still consistent with our previous results from all 15 studies.

## Discussion

This meta-analysis of 3 RCTs and 12 retrospective studies including 54107 patients comparing the ACDF with PCF showed that PCF had satisfactory clinical outcomes and comparative complication rate for cervical radiculopathy. However, a higher revision rate was also found in the PCF group.

### Clinical outcomes

ACDF is a commonly performed surgical procedure for patients suffering from the neck and arm pain due to cervical radiculopathy. The clinical outcome of this procedure is good or excellent in the majority of cases and is hence thought to be the “gold standard” surgical treatment for degenerative cervical diseases [31, 32]. From 2010 to 2016, the data from a national database showed significantly more patients diagnosed with cervical radiculopathy underwent ACDF (more than 70%) compared with the number of patients who underwent cervical disc replacement (CDR) and PCF [17]. ACDF remains the most common surgical procedure for cervical radiculopathy, and its utilization has remained consistent during recent years. A randomized controlled trial by Engquist et al. reported that ACDF can achieve great and rapid improvement in reducing neck disability, health state, neck pain, and arm pain [4]. These scores continued to improve between the 2-year and 5- to 8-year follow-ups, whereas non-surgically treated patients showed no further improvement.

PCF can directly decompress the nerve root and achieve successful patient symptom resolution and improvement in patient quality of life [33]. A telephone interview of 338 patients who underwent PCF with a mean follow-up of 10 years reported that approximately 90% of interviewees had improved pain, weakness, or function and 93% of patients were able to return to work after PCF [30]. However, the narrow indication of PCF for unilateral upper extremity radiculopathy due to posterolateral or foraminal disk herniation or osteophyte, and the drawback of dorsal muscle dissection resulted from PCF mainly limited its usage [33, 34]. Recently, a minimally invasive surgery (MI-PCF) that uses a tubular retractor or a full-endoscopic system has been performed for the patients with cervical radiculopathy. The MI-PCF can share the advantage of open PCF, which maintain the cervical mobility while spare the problems related to extensive muscle dissection [34, 35]. Rutten et al. conducted a perspective study regarding the full-endoscopic PCF, and the 2-year results revealed that MI-PCF is a sufficient and safe procedure. Ninety-three percent of participants reported subjective satisfaction and would again undergo the same procedure. A meta-analysis has concluded that patients with symptomatic cervical radiculopathy can be effectively managed with either an open or MI approach, and the clinical outcomes between the two groups were equivalent [11].

Various studies have tried to compare the ACDF with PCF for the treatment of cervical radiculopathy, but no consensus has been reached. Although many of them suggested the clinical results of PCF are equal to the conventional ACDF [20, 26, 28], there are still some other studies that had a different conclusion. Scholz et al. [19] reported a better overall outcome and greater relief of radicular and neck pain in the PCF group in a retrospective study. On the other hand, Herkowitz et al. [25] recommended ACDF for the surgical management of anterolateral soft disc herniation because it can provide better long-term improvement than PCF. Our meta-analysis synthesized comparative data of these 2 surgical treatments and revealed that the pain and functional outcomes including VAS-neck, VAS-arm, and NDI were similar between treatment groups. Our results are consistent with the results of a systematic review published in 2016, which used only qualitative analysis without statistical combination [36]. The systematic review also concluded the PCF was as safe and effective as the ACDF for the treatment of cervical radiculopathy. The two aforementioned meta-analysis comparing MI-PCF and ACDF also concluded that MI-PCF may be utilized as an effective alternative to ACDF for unilateral radiculopathy [12, 13].

### Complications and reoperations

According to our meta-analysis, the complication rate was equivalent between treatment groups. In a retrospective study including 1015 patients who underwent ACDF, postoperative dysphagia, hematoma, and unilateral recurrent laryngeal nerve palsy were the most common complications, while esophageal perforation was the most serious one [8]. Urinary complications and wound complications were reported to be the most common complications following single-level PCF in a recent large sample review [37]. They also found out that patients who received PCF in an inpatient setting showed significantly higher rates of wound complications, infection, acute respiratory failure, and urinary complications than patients who receive outpatient PCF. Increased incidence of surgical and medical complications in the inpatient PCF group may be attributed to the fact that inpatient patients have more comorbidities than outpatient patients. In addition, outpatient surgery could theoretically reduce the risk of nosocomial infections. Witiw et al. reviewed the data of more than 50,000 patients who underwent ACDF or PCF for single-level cervical radiculopathy from a US commercial health insurance claims database spanning 2003 to 2014 [16]. They revealed that vascular injury, postoperative dysphagia/dysphonia, cerebrospinal fluid (CSF) leak, and deep venous thrombosis occurred in a significantly higher proportion of patients undergoing ACDF, while postoperative wound infection was significantly higher in the PCF cohort.

The overall reoperation rate was 7.59% in the PCF group, statistically higher than that in the ACDF group (4.05%). The reoperation cases in the PCF group were mostly operated at the same level due to persistent or reappearing radicular pain, whereas adjacent segment disease (ASD) was the most common reason for performing reoperation following ACDF. A retrospective long-term analysis in 374 patients undergoing 409 anterior cervical fusions reported a relatively consistent incidence of ASD of 3% per year [6]. Symptomatic ASD may affect more than one fourth of all patients within 10 years after ACDF. On the other hand, some surgeons believe PCF could decrease the incidence of ASD. Clarke et al. [38] retrospectively analyzed data obtained from 303 patients who underwent PCF for cervical radiculopathy. The rates of ASD were estimated to be 0.9% per year and 6.7% per 10 years following PCF. Skovrlj et al. [39] calculated a similar rate of 0.9% per year for the cases of symptomatic ASD requiring reoperation following PCF. Although we could not do a quantitative comparative analysis based on the included studies, the reported rates of symptomatic ASD following PCF was indeed less than that following ACDF. The significantly increased range of motion adjacent to the operated segment after ACDF compared with PCF may contribute to the higher rate of ASD [9, 10].

### Perioperative outcomes

PCF was associated with shorter operation time and shorter length of hospital stay according to our analysis. Compared to ACDF, a significantly greater proportion of patients in the PCF group underwent outpatient surgery or 1-day hospitalization after surgery [16]. Mesregah et al. reported that outpatient single-level PCF was associated with a lower rate of perioperative medical and surgical complications compared with inpatient PCF, and PCF in the outpatient setting was suggested to be a safe procedure for the [37].

Total hospital cost in the PCF group was significantly less than the ACDF group, the reason of which may be attributable to a greater proportion of PCF procedures occurring in the outpatient setting and an avoidance of the need for surgical implants [16, 27]. Given the comparable clinical outcomes for either procedure, PCF was considered to be more cost-effective than ACDF [22, 30].

## Limitation

Our meta-analysis has certain limitations as follows. First, apart from the 3 RCTs which had small sample sizes, the majority of our included studies were non-randomized studies. Although the included studies were of relatively high quality according to the NOS assessment, we must acknowledge that further randomized controlled trials with high quality and more patients should be performed. Second, the majority of the cases in this meta-analysis were from Witiw et al.’s study [17] and the data of their study was obtained from a commercial health insurance database, which was different from other studies. Although the sensitivity analysis revealed that the relevant results were consistent and stable, the potential heterogeneity raised from this study should be also aware. Third, rare studies reported the data of radiographic outcomes; hence, we could not compare the two treatment groups on a radiographic level.

## Conclusion

Among patients with single-level unilateral cervical radiculopathy, PCF has comparable effectiveness and complication rate compared with ACDF. It seems that PCF is a sufficient alternative procedure with shorter operation time, shorter length of hospital stay, and less total hospital cost for the treatment of cervical radiculopathy. However, the higher reoperation rate following PCF should be also taken into consideration.

## Data Availability

The datasets used and analyzed during the current study are available from the corresponding author on reasonable request.

## Abbreviations

ACDF: Anterior cervical discectomy and fusion
ASD: Adjacent segment disease
CDR: Cervical disc replacement
CI: Confidence interval
CSF: Cerebrospinal fluid
MI: Minimally invasive
NDI: Neck Disability Index
NOS: Newcastle-Ottawa Scale
OR: Odds ratios
PCF: Posterior cervical foraminotomy
RCT: Randomized controlled trial
VAS: Visual analog scale
WMD: Weighted mean difference

## References

[1] Bono CM, Ghiselli G, Gilbert TJ, Kreiner DS, Reitman C, Summers JT, Baisden JL, Easa J, Fernand R, Lamer T, Matz PG, Mazanec DJ, Resnick DK, Shaffer WO, Sharma AK, Timmons RB, Toton JF, North American Spine S (2011). An evidence-based clinical guideline for the diagnosis and treatment of cervical radiculopathy from degenerative disorders. Spine J.

[2] Persson LC, Moritz U, Brandt L, Carlsson CA (1997). Cervical radiculopathy: pain, muscle weakness and sensory loss in patients with cervical radiculopathy treated with surgery, physiotherapy or cervical collar. A prospective, controlled study. Eur Spine J.

[3] Sampath P, Bendebba M, Davis JD, Ducker T. Outcome in patients with cervical radiculopathy. Prospective, multicenter study with independent clinical review. Spine (Phila Pa 1976) 24:591-597. 1999. 10.1097/00007632-199903150-00021. 10.1097/00007632-199903150-00021 10101827

[4] Engquist M, Lofgren H, Oberg B, Holtz A, Peolsson A, Soderlund A, Vavruch L, Lind B (2017). A 5- to 8-year randomized study on the treatment of cervical radiculopathy: anterior cervical decompression and fusion plus physiotherapy versus physiotherapy alone. J Neurosurg Spine.

[5] Hauerberg J, Kosteljanetz M, Boge-Rasmussen T, Dons K, Gideon P, Springborg JB, Wagner A (2008). Anterior cervical discectomy with or without fusion with ray titanium cage: a prospective randomized clinical study. Spine (Phila Pa 1976).

[6] Hilibrand AS, Carlson GD, Palumbo MA, Jones PK, Bohlman HH (1999). Radiculopathy and myelopathy at segments adjacent to the site of a previous anterior cervical arthrodesis. J Bone Joint Surg Am.

[7] Fraser JF, Hartl R (2007). Anterior approaches to fusion of the cervical spine: a metaanalysis of fusion rates. J Neurosurg Spine.

[8] Fountas KN, Kapsalaki EZ, Nikolakakos LG, Smisson HF, Johnston KW, Grigorian AA, Lee GP, Robinson JS (2007). Anterior cervical discectomy and fusion associated complications. Spine (Phila Pa 1976).

[9] Cho TG, Kim YB, Park SW (2014) Long term effect on adjacent segment motion after posterior cervical foraminotomy. Korean J Spine 11:1-6. doi: 10.14245/kjs.2014.11.1.1. 10.14245/kjs.2014.11.1.1 PMC4040637 24891864

[10] Lin GX, Rui G, Sharma S, Kotheeranurak V, Suen TK, Kim JS (2019). Does the neck pain, function, or range of motion differ after anterior cervical fusion, cervical disc replacement, and posterior cervical foraminotomy?. World Neurosurg.

[11] McAnany SJ, Kim JS, Overley SC, Baird EO, Anderson PA, Qureshi SA (2015). A meta-analysis of cervical foraminotomy: open versus minimally-invasive techniques. Spine J.

[12] Sahai N, Changoor S, Dunn CJ, Sinha K, Hwang KS, Faloon M, Emami A. Minimally Invasive posterior cervical foraminotomy as an alternative to anterior cervical discectomy and fusion for unilateral cervical radiculopathy: a systematic review and meta-analysis. Spine (Phila Pa 1976). 2019. 10.1097/BRS.0000000000003156. 10.1097/BRS.0000000000003156 31343619

[13] Gutman G, Rosenzweig DH, Golan JD (2018) Surgical treatment of cervical radiculopathy: meta-analysis of randomized controlled trials. Spine (Phila Pa 1976) 43:E365-E372. doi: 10.1097/BRS.0000000000002324 [doi]. 10.1097/BRS.0000000000002324 28700452

[14] Wells G, Shea B, O'Connell D, Peterson J, Welch V, Losos M, Tugwell P The Newcastle-Ottawa Scale (NOS) for assessing the quality of nonrandomised studies in meta-analyses. http://wwwohrica/programs/clinical_epidemiology/oxfordasp.

[15] Higgins J, Green S (2011) Cochrane Handbook for Systematic Reviews of Interventions Version 5.1.0 [updated March 2011]. The Cochrane Collaboration: available from www.cochrane-handbook.org.

[16] Witiw CD, Smieliauskas F, O'Toole JE, Fehlings MG, Fessler RG (2019). Comparison of Anterior cervical discectomy and fusion to posterior cervical foraminotomy for cervical radiculopathy: utilization, costs, and adverse events 2003 to 2014. Neurosurgery.

[17] Mok JK, Sheha ED, Samuel AM, McAnany SJ, Vaishnav AS, Albert TJ, Gang CH, Qureshi S (2019) Evaluation of current trends in treatment of single-level cervical radiculopathy. Clin Spine Surg 32:E241-E245. doi: 10.1097/BSD.0000000000000796 [doi]. 10.1097/BSD.0000000000000796 30762836

[18] Foster MT, Carleton-Bland NP, Lee MK, Jackson R, Clark SR, Wilby MJ (2019) Comparison of clinical outcomes in anterior cervical discectomy versus foraminotomy for brachialgia. Br J Neurosurg 33:3-7. doi: 10.1080/02688697.2018.1527013 [doi]. 10.1080/02688697.2018.1527013 30450995

[19] Scholz T, Geiger MF, Mainz V, Blume C, Albanna W, Clusmann H, Muller A (2018) Anterior cervical decompression and fusion or posterior foraminotomy for cervical radiculopathy: results of a single-center series. J Neurol Surg A Cent Eur Neurosurg 79:211-217. doi: 10.1055/s-0037-1607225 [doi]. 10.1055/s-0037-1607225 29132169

[20] Alvin MD, Lubelski D, Abdullah KG, Whitmore RG, Benzel EC, Mroz TE (2016) Cost-utility analysis of Anterior Cervical Discectomy and Fusion With Plating (ACDFP) versus Posterior Cervical Foraminotomy (PCF) for patients with single-level cervical radiculopathy at 1-year follow-up. Clin Spine Surg 29:E67-72. doi: 10.1097/BSD.0000000000000099 [doi] 201603000-00012 [pii]. 10.1097/BSD.0000000000000099 26889994

[21] Selvanathan SK, Beagrie C, Thomson S, Corns R, Deniz K, Derham C, Towns G, Timothy J, Pal D (2015) Anterior cervical discectomy and fusion versus posterior cervical foraminotomy in the treatment of brachialgia: the Leeds spinal unit experience (2008-2013). Acta Neurochir (Wien) 157:1595-1600. doi: 10.1007/s00701-015-2491-8 [doi] 1007/s00701-015-2491-8 [pii]. 10.1007/s00701-015-2491-8 26144567

[22] Tumialan LM, Ponton RP, Gluf WM (2010) Management of unilateral cervical radiculopathy in the military: the cost effectiveness of posterior cervical foraminotomy compared with anterior cervical discectomy and fusion. Neurosurg Focus 28:E17. doi: 10.3171/2010.1.FOCUS09305 [doi]. 10.3171/2010.1.FOCUS09305 20568933

[23] Korinth MC, Kruger A, Oertel MF, Gilsbach JM (2006) Posterior foraminotomy or anterior discectomy with polymethyl methacrylate interbody stabilization for cervical soft disc disease: results in 292 patients with monoradiculopathy. Spine (Phila Pa 1976) 31:1207-1214; discussion 1215-1206. doi: 10.1097/01.brs.0000217604.02663.59 [doi] -200605150-00006 [pii]. 10.1097/01.brs.0000217604.02663.59 16688033

[24] Wirth FP, Dowd GC, Sanders HF, Wirth C (2000) Cervical discectomy. A prospective analysis of three operative techniques. Surg Neurol 53:340-346; discussion 346-348. doi: S0090-3019(00)00201-9 [pii]. 10.1016/s0090-3019(00)00201-9 10825519

[25] Herkowitz HN, Kurz LT, Overholt DP (1990). Surgical management of cervical soft disc herniation. A comparison between the anterior and posterior approach. Spine (Phila Pa 1976).

[26] Dunn C, Moore J, Sahai N, Issa K, Faloon M, Sinha K, Hwang KS, Emami A (2018) Minimally invasive posterior cervical foraminotomy with tubes to prevent undesired fusion: a long-term follow-up study. J Neurosurg Spine 29:358-364. doi: 10.3171/2018.2.SPINE171003 [doi] 2.SPINE171003 [pii]. 10.3171/2018.2.SPINE171003 29957145

[27] Mansfield HE, Canar WJ, Gerard CS, O'Toole JE (2014) Single-level anterior cervical discectomy and fusion versus minimally invasive posterior cervical foraminotomy for patients with cervical radiculopathy: a cost analysis. Neurosurg Focus 37:E9. doi: 10.3171/2014.8.FOCUS14373 [doi]. 10.3171/2014.8.FOCUS14373 25491887

[28] Ruetten S, Komp M, Merk H, Godolias G (2008) Full-endoscopic cervical posterior foraminotomy for the operation of lateral disc herniations using 5.9-mm endoscopes: a prospective, randomized, controlled study. Spine (Phila Pa 1976) 33:940-948. doi: 10.1097/BRS.0b013e31816c8b67 [doi] -200804200-00003 [pii]. 10.1097/BRS.0b013e31816c8b67 18427313

[29] Moher D, Liberati A, Tetzlaff J, Altman DG, Group P (2009). Preferred reporting items for systematic reviews and meta-analyses: the PRISMA statement. Bmj.

[30] Church EW, Halpern CH, Faught RW, Balmuri U, Attiah MA, Hayden S, Kerr M, Maloney-Wilensky E, Bynum J, Dante SJ, Welch WC, Simeone FA (2014). Cervical laminoforaminotomy for radiculopathy: symptomatic and functional outcomes in a large cohort with long-term follow-up. Surg Neurol Int.

[31] Nasca RJ (2009) Cervical radiculopathy: current diagnostic and treatment options. J Surg Orthop Adv 18:13-18. doi: 18-1-3.pdf?T = open_article,50021211 [pii]. 19327260

[32] Mazas S, Benzakour A, Castelain JE, Damade C, Ghailane S, Gille O (2019) Cervical disc herniation: which surgery? Int Orthop 43:761-766. doi: 10.1007/s00264-018-4221-3 [doi] 1007/s00264-018-4221-3 [pii]. 10.1007/s00264-018-4221-3 30411247

[33] Dodwad SJ, Dodwad SN, Prasarn ML, Savage JW, Patel AA, Hsu WK (2016) Posterior cervical foraminotomy: indications, technique, and outcomes. Clin Spine Surg 29:177-185. doi: 10.1097/BSD.0000000000000384 [doi]. 10.1097/BSD.0000000000000384 27187617

[34] Papavero L, Kothe R (2018) Minimally invasive posterior cervical foraminotomy for treatment of radiculopathy: an effective, time-tested, and cost-efficient motion-preservation technique. Oper Orthop Traumatol 30:36-45. doi: 10.1007/s00064-017-0516-6 [doi] 1007/s00064-017-0516-6 [pii]. 10.1007/s00064-017-0516-6 28929274

[35] Coric D, Adamson T (2008) Minimally invasive cervical microendoscopic laminoforaminotomy. Neurosurg Focus 25:E2. doi: 10.3171/FOC/2008/25/8/E2 [doi]. 10.3171/FOC/2008/25/8/E2 18673049

[36] Liu WJ, Hu L, Chou PH, Wang JW, Kan WS (2016) Comparison of anterior cervical discectomy and fusion versus posterior cervical foraminotomy in the treatment of cervical radiculopathy: a systematic review. Orthop Surg 8:425-431. doi: 10.1111/os.12285 [doi]. 10.1111/os.12285 PMC6584082 28032703

[37] Mesregah MK, Chantarasirirat K, Formanek B, Buser Z, Wang JC. Perioperative complications of inpatient and outpatient single-level posterior cervical foraminotomy: a comparative retrospective study. Spine J. 2019. 10.1016/j.spinee.2019.08.010. 10.1016/j.spinee.2019.08.010 31442615

[38] Clarke MJ, Ecker RD, Krauss WE, McClelland RL, Dekutoski MB (2007). Same-segment and adjacent-segment disease following posterior cervical foraminotomy. J Neurosurg Spine.

[39] Skovrlj B, Gologorsky Y, Haque R, Fessler RG, Qureshi SA (2014). Complications, outcomes, and need for fusion after minimally invasive posterior cervical foraminotomy and microdiscectomy. Spine J.

